# First draft genome assembly and identification of SNPs from hilsa shad ( *Tenualosa ilisha*) of the Bay of Bengal

**DOI:** 10.12688/f1000research.18325.1

**Published:** 2019-03-22

**Authors:** Md. Bazlur Rahman Mollah, Mohd Golam Quader Khan, Md Shahidul Islam, Md Samsul Alam

**Affiliations:** 1 Poultry Biotechnology and Genomics Laboratory, Department of Poultry Science, Bangladesh Agricultural University, Mymensingh, 2202, Bangladesh; 2 Department of Fisheries Biology and Genetics, Bangladesh Agricultural University, Mymensingh, 2202, Bangladesh; 3 Department of Biotechnology, Bangladesh Agricultural University, Mymensingh, 2202, Bangladesh

**Keywords:** Hilsa, anadromous, Bay of Bengal, whole genome, SNP

## Abstract

**Background**: Hilsa shad ( *Tenualosa ilisha*), a widely distributed migratory fish, contributes substantially to the economy of Bangladesh. The harvest of hilsa from inland waters has been fluctuating due to anthropological and climate change-induced degradation of the riverine habitats.  The whole genome sequence of this valuable fish could provide genomic tools for sustainable harvest, conservation and productivity cycle maintenance. Here, we report the first draft genome of *T. ilisha* from the Bay of Bengal, the largest reservoir of the migratory fish.

**Methods**: A live specimen of *T. ilisha* was collected from the Bay of Bengal. The whole genome sequencing was performed by the Illumina HiSeqX platform (2 × 150 paired end configuration). We assembled the short reads using SOAPdenovo2 genome assembler and predicted protein coding genes by AUGUSTUS. The completeness of the *T. ilisha* genome assembly was evaluated by BUSCO (Benchmarking Universal Single Copy Orthologs). We identified single nucleotide polymorphisms (SNPs) by calling them directly from unassembled sequence reads using discoSnp++.

**Results**: We assembled the draft genome of 710.28 Mb having an N50 scaffold length of 64157 bp and GC content of 42.95%. A total of 37,450 protein coding genes were predicted of which 29,339 (78.34%) were annotated with other vertebrate genomes. We also identified 792,939 isolated SNPs with transversion:transition ratio of 1:1.8. The BUSCO evaluation showed 78.1% completeness of this genome.

**Conclusion**s: The genomic data generated in this study could be used as a reference to identify genes associated with physiological and ecological adaptations, population connectivity, and migration behaviour of this biologically and economically important anadromous fish species of the Clupeidae family.

## Introduction

Hilsa shad ( *Tenualosa ilisha*) is a migratory fish of the Clupeidae family. It is distributed from the South China Sea and through the Bay of Bengal to the Persian Gulf. The riverine habitats of this fish include the Padma, Jamuna, Meghna, Karnaphuli and the coastal rivers of Bangladesh, the Tigris and Euphrates of Iran and Iraq, the Indus of Pakistan, the rivers of Eastern and Western India and the Irrawaddy of Myanmar ( Freyhof, 2014; Pillay & Rosa, 1963). Ecologically, three different types of hilsa shad have been recognized in Bangladesh waters such as anadromous, potamodromous and marine ( Milton, 2010).

Hilsa is the most popular and economically important food fish in Bangladesh, contributing 12% of the total fish production and 1.15% of GDP. Of its world catch, 60% amounting to 0.5 million metric tons, comes from Bangladesh ( DoF, 2018). Though the overall production of hilsa increased over the years, gross decline in productions were evident from inland waters. A number of factors such as overexploitation, siltation in river beds, decrease in water ﬂow from upstream and fragmentation of the rivers are attributed to this fluctuation in productivity ( Ahsan *et al.,* 2014). To enhance hilsa production, programs like the establishment of sanctuaries, restrictions on the use of fishing equipment and a ban on fishing in certain periods of the year to protect parent and juvenile fish have been initiated. It is, however, very important that the management activities be matched with the biological features of the fish for their effectiveness. Inconclusive information about the management units and the level of connectivity amongst them is considered as the major constraint in formulating appropriate hilsa management plans.

There are controversies regarding the number of hilsa stocks in Bangladesh waters. Studies involving morphological and genetic analyses using allozyme, Random Amplification of Polymorphic DNA (RAPD) and mtDNA-restriction fragment length polymorphism (RFLP) markers proved to be insufficient in resolving the stock disputes of this species ( Ahmed *et al.,* 2004; Mazumder & Alam, 2009; Salini *et al.,* 2004). DNA markers derived from whole genome sequencing are more efficient to define management units, quantify the extent of adaptive divergence and connectivity among stocks, and to perform mixed-stock analysis ( Martinez Barrio *et al.,* 2016; Figueras *et al.,* 2016; Machado *et al.,* 2018). Single nucleotide polymorphic (SNP) markers allow whole genome coverage and high levels of automation. Conventionally, SNP markers are developed by comparing nucleotide sequences with a reference genome. Recent advancement in generating SNPs from reference-free whole genome sequences accelerated identification of SNPs from non-model organisms. Although hilsa is a very important fish biologically and economically, it lacks a reference genome and genomic resources, imposing a severe bottleneck to understand its physiological and ecological requirements. Therefore, we performed whole genome sequencing ( Mollah *et al.,* 2018), constructed a draft genome assembly and identified SNPs from *T. ilisha* of the Bay of Bengal.

## Methods

### Sample collection and genomic DNA extraction

**Figure 1.**
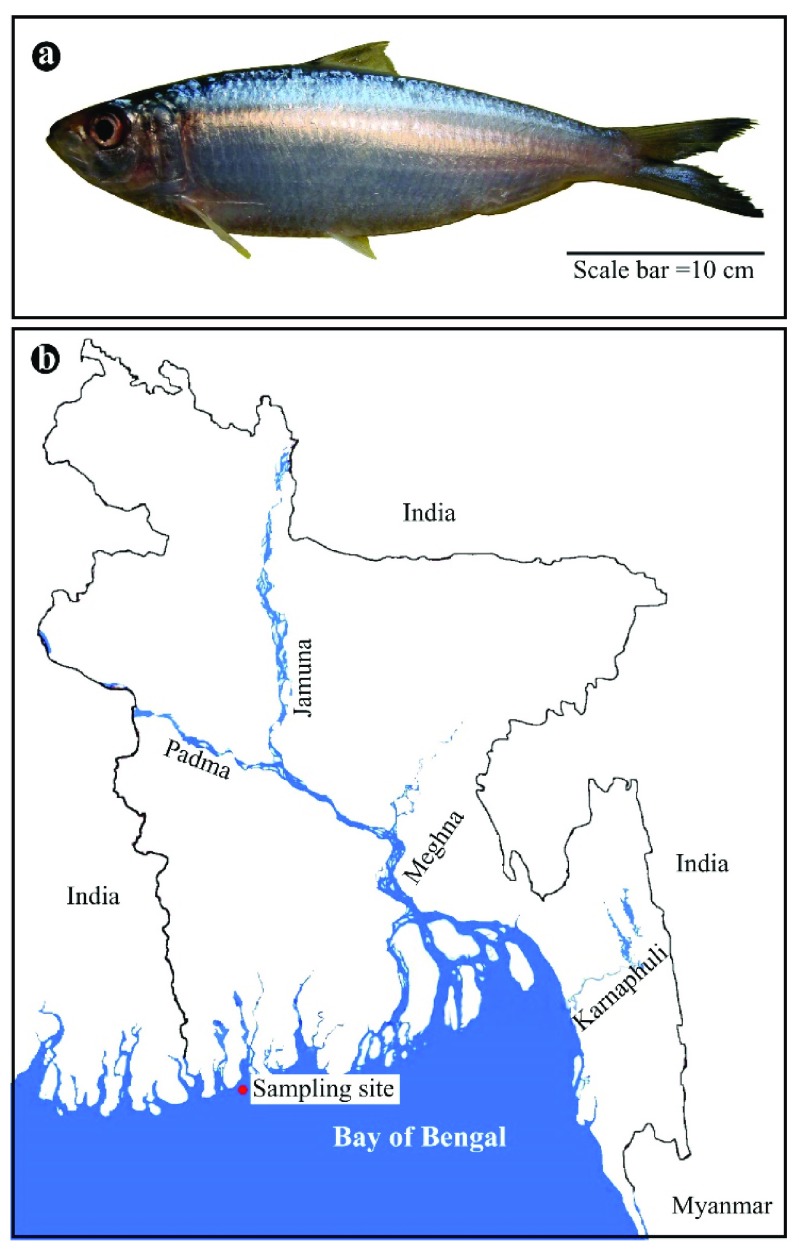
The experimental fish and its collection site. Photograph of a *T. ilisha* specimen ( **a**) and a map of Bangladesh showing the sampling site (21.981753 N 90.305556 E) ( **b**).

**Figure 2.**
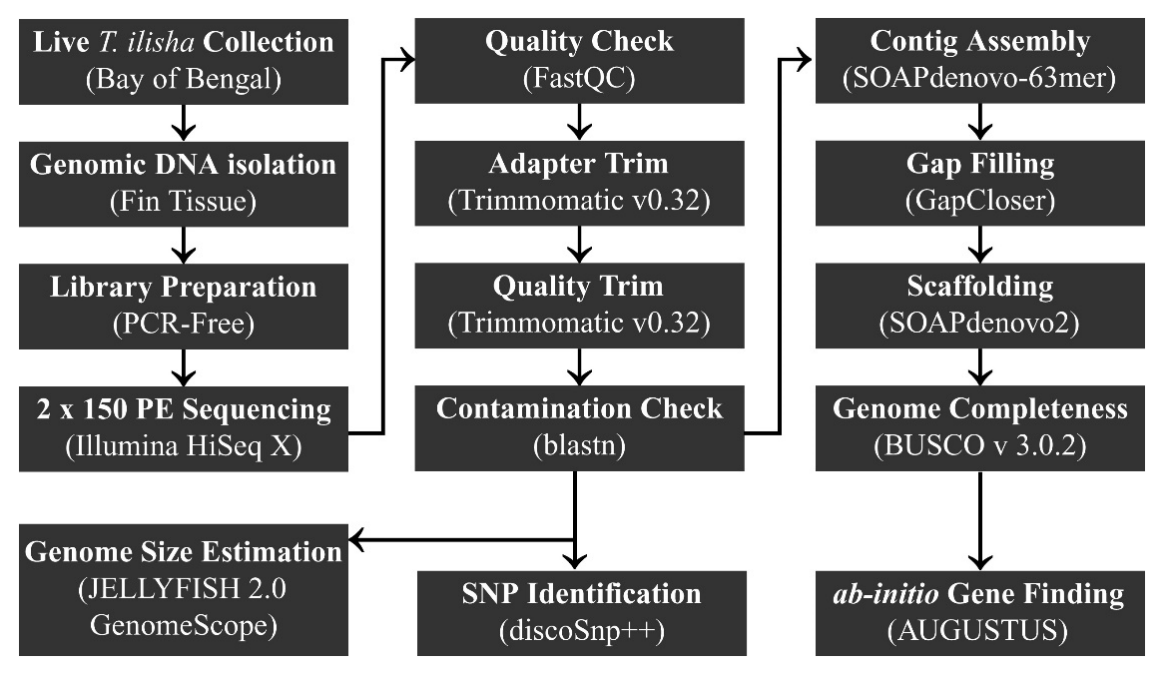
Methodology outline. Schematic diagram illustrating the methodology of whole genome sequencing, *de novo* assembly and identification of SNPs in *T. ilisha* from the Bay of Bengal.

We captured ten *T. ilisha* specimens from the seashore of the Bay of Bengal (21.981753 N 90.305556 E) ( Figure 1). All efforts were made to ameliorate harm to the fish by using a seine net of appropriate mesh size (20 mm) to avoid any physical injuries and suffocation. Due to the nature of this species, the fish died immediately after taking them out of the water. Dorsal and caudal fin tissues were immediately clipped on board from a dead female fish (560.42g) and preserved in 96% ethanol. The fish were handled according to the guidelines of the Animal Welfare and Ethical Committee (AWEC) of Bangladesh Agricultural University. The genomic DNA was isolated using the standard phenol:chlorofom:isoamyl alcohol method ( Sambrook & Russell, 2001). DNA purity was evaluated using a NanoDrop 2000 Spectrophotometer (ThermoFisher Scientific, cat # ND-2000) and 0.8% agarose gel electrophoresis. DNA was quantified using Qubit 2.0 Fluorometer and Qubit dsDNA HS Assay Kit (ThermoFisher Scientific, Cat. # Q32851) and used to systematically generate the whole genome sequence data ( Figure 2).

### PCR-free DNA library preparation and sequencing

For sequencing, a PCR-free DNA library was prepared using the Illumina TruSeq DNA PCR-free Library Preparation Kit (Cat. # 20015963), following the manufacturer’s recommendations (Illumina, CA, USA). The library was fragmented, size was selected following the 350 bp insert size scheme and validated using TapeStation (2100 Bioanalyzer,Agilent Technologies, CA, USA). The DNA library was quantified using a Qubit 2.0 Fluorometer as well as by real time PCR (ABI 7500, Applied Biosystems, CA, USA) using the KAPA Library Quantification Kit (Cat. # KK4824) following the manufacturer’s standard protocol with the primer pair Primer 1: 5'-AAT GAT ACG GCG ACC ACC GA-3' Primer 2: 5'-CAA GCA GAA GAC GGC ATA CGA-3'. The PCR condition was followed as initial denaturation at 95°C for 5 min followed by 35 cycles (denaturation at 95°C for 30 sec, annealing/extension/data acquisition at 60°C for 45 sec) and melt curve analysis at 65 – 95°C. Sequencing was performed on the Illumina HiSeqX instrument according to the manufacturer’s instructions. The library was sequenced using a 2× 150 paired-end (PE) configuration (GENEWIZ, LLC. South Plainfield, NJ, USA).

### DNA sequence processing and genome size estimation

The raw reads were filtered based on quality and length using Trimmomatic-0.32 ( Bolger *et al.,* 2014) after evaluating with FastQC v. 0.11.8 ( Andrews, 2018) as follows: i) removal of adaptor sequences; ii) removal of read pairs from either ends when the base quality was <20; iii) trimming low quality fragments at both ends of the reads within a window size of 4 bp and an average quality threshold of 15; iv) removal of read pairs having <75 nucleotides. Jellyfish v. 2.2.6 ( Marçais & Kingsford, 2011) was used to obtain a separate frequency distribution of three different high occurring kmers (21, 31 and 33) in the raw HiSeq sequence reads, and the histograms were uploaded to GenomeScope for estimating genome size, repeat content, repeat length, unique length and heterozygosity following kmer-based statistical approaches ( Vurture *et al.,* 2017).

### Genome assembly, genome quality evaluation and annotation

We assembled the short reads using SOAPdenovo2 genome assembler ( Luo *et al.,* 2012), developed specifically for use with next-generation short-read sequences. SOAPdenovo2 uses the de Bruijn graph algorithm. We tested several kmers to assemble the *T. ilisha* genome and finally selected the assembly with a kmer of 33. The completeness of the *T. ilisha* genome assembly was evaluated by BUSCO (Benchmarking Universal Single Copy Orthologs) ( Simão *et al.,* 2015). For BUSCO analysis (-m geno –sp zebrafish settings), the genome was searched against the Actinopterygii database (actinopterygii_odb9), which was constructed from 20 fish species consisting of 4584 orthologs. AUGUSTUS *ab initio* gene prediction was performed to predict protein-coding genes. The protein sequences of fish species and other vertebrates, including *Rhincodon typus*, *Cyprinus carpio*, *Takifugu rubripes*, *Salmo salar*, *Mus musculus* and *Homo sapiens*, were downloaded from the NCBI non-redundant protein sequences (nr) database ( Table 3) and aligned against the *T. ilisha* genome using BLASTP ( Altschul *et al.,* 1997).

### Reference-free detection of isolated SNPs

We used discoSnp++ v2.2.10 ( Uricaru *et al.,* 2015) with default parameters for reference-free detection of isolated SNPs (SNPs not flanked by other SNPs, Indels or structural variants) by calling SNPs directly from sequence reads without a reference genome. This method identifies isolated SNPs from the sequences of two homologous chromosomes within a single individual. 

## Results and discussion

**Figure 3.**
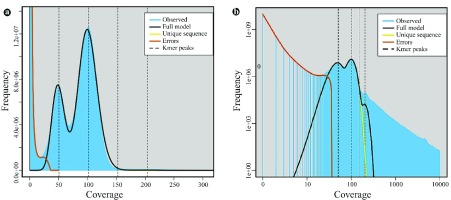
GenomeScope kmer profile plot of the *T. ilisha*. Dataset show the fit of the GenomeScope model (black) based on 33-kmers in Illumina HiSeq sequence reads, max kmer coverage at 300× ( **a**) and 10000× coverage ( **b**).

The estimated haploid genome size of *T. ilisha* ranged from 649.48 to 660.73 Mb. We observed heterozygosity and a repeat peak ( Figure 3), with an estimated heterozygosity of 0.579 to 0.660% and repeats of 8.30 to 13.57% ( Table 1). We assembled the draft genome of 710.28 Mb, having an N50 scaffold length of 64157 bp and a GC content of ~43% ( Table 2). The whole genome assembly of a notable Clupeid fish, the Atlantic herring, based on short reads (170 bp to 20 kb inserts) was 808 Mb with a scaffold N50 of 1.84 Mb and GC content of 44%, with repetitive elements making up 31% of the assembly ( Martinez Barrio *et al.,* 2016). The genome size of another important Clupeid fish, the European sardine ( *Sardina pilchardus*), was estimated to be 655-850 Mb ( Machado *et al.,* 2018) and 935-950Mb ( Louro *et al.,* 2018).

**Table 1.** Properties of *T. ilisha* genome estimated at three different kmers ^1^.

Properties	Kmer 21		Kmer 31		Kmer 33	
	min	max	min	max	min	max
Genome Haploid Length (bp)	649,475,766	649,949,877	659,441,333	659,890,585	660,289,984	660,728,342
Heterozygosity (%)	0.654	0.660	0.590	0.594	0.579	0.583
Genome Repeat Length (bp)	88,141,621	88,205,963	57,904,063	57,943,510	54,791,371	54,827,747
Genome Unique Length (bp)	561,334,145	561,743,914	601,537,270	601,947,074	605,498,612	605,900,595
Read Error Rate (%)	0.527		0.477		0.468	

^1^Kmers are unique subsequences of a sequence of length k. The estimated genome size varies according to kmer value. The estimated haploid genome lengths obtained from kmer 31 and kmer 33 are very close.

**Table 2.** Contig and scaffold properties of *T. ilisha* genome.

Contig			Scaffold	
Parameters	Value	%	Parameters	Value
Read pairs	769,262,291	-	Scaffold Number	100181
Contig Number	1724390	-	Mean Scaffold size	7090
Mean Contig Size	378	-	Longest Scaffold	832708
Median Contig Size	209	-	Shortest Scaffold	200
Longest Contig	27277	-	N10	254367
Shortest Contig	100	-	N30	118059
Contig >100bp	1704389	98.84	N50	64157
Contig >500bp	335422	19.45	N70	26438
Contig >1K	121379	7.04	N90	4991
Contig >10K	58	0.00	N count	96164104
Contig N50 (bp)	594	-	Assembled Genome Size (bp)	710279582
G+C content %	-	43.01	G+C content (%)	42.95

**Table 3.** Significant matches (blastp e = 0.001) of *T. ilisha* genes with other vertebrates.

Species	No. of matches	% match
*Rhincodon typus*	27062	72.26
*Cyprinus carpio*	28373	75.76
*Takifugu rubripes*	28325	75.63
*Salmo salar*	29339	78.34
*Mus musculus*	26480	70.71
*Homo sapiens*	27497	73.42

The assembled *T. ilisha* genome was searched for BUSCO analysis against the Actinopterygii database, consisting of 4,584 orthologs constructed from 20 fish species. We found 3,578 complete (C: 78.1%), 3,456 complete and single-copy (S: 75.4%), 122 complete and duplicated (D: 2.7%), 351 fragmented (F: 7.7%) and 655 missing BUSCOs (M: 14.2%). These results suggest higher completeness of the *T. ilisha* genome assembly of the Bay of Bengal. The BUSCO analysis of its closely related species, the European sardine, showed 84% genome completeness ( Louro *et al.,* 2018). The completeness of genome assembly may depend on the sequencing platform used. For example, 92.3% BUSCO completeness was obtained using only the Illumina reads compared to 94.2% completeness from the Illumina + Nanopore reads in the Murray cod ( *Maccullochella peelii*) ( Austin *et al.,* 2017).

AUGUSTUS *ab initio* gene prediction was performed to predict protein-coding genes. We found 37,450 protein coding genes from the assembled *T. ilisha* genome ( Mollah *et al.,* 2019a). To annotate the proteins, predicted amino acid sequences of *T. ilisha* were aligned against the NCBI non-redundant protein sequences (nr) database of other vertebrates ( Table 3) using BLASTP. Among the five vertebrate genomes compared, a minimum of 70.71% genes of *T. ilisha* was annotated by *Mus musculus* and a maximum of 78.34% by *Salmo salar* ( Table 3). The numbers of predicted protein coding genes in two other important Clupeids, the Atlantic herring and the European sardine, were 23,336 and 29,408, respectively ( Martinez Barrio *et al.,* 2016; Louro *et al.,* 2018).

**Figure 4.**
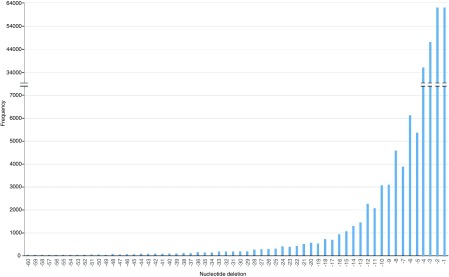
Distribution of Indels in the *T. ilisha* genome. The indel values ranged from 1 to 60 nucleotides. It shows that the frequency of indels decreased with the increase in size.

We identified a total of 792,939 isolated SNPs in *T. ilisha* genome, of which 510,251 were transitions and 282,688 were transversions ( Table 4) ( Mollah *et al.,* 2019b). We also detected 155,574 indels ranging in sizes from 1 to 60 nucleotides ( Figure 4). A total of 5.3 million raw SNPs in the Atlantic stock and 5.2 million SNPs in the Baltic stock of the Atlantic herring were detected by Feng *et al.* (2017). In contrast, Louro *et al.* (2018) identified a total of 2.3 million filtered heterozygous SNPs in the European sardine. Since there is no high quality reference genome available in *T. ilisha*, we used discoSNP++ because of its nobility and efficiency in detection of SNPs from unassembled genome sequences. Uricaru *et al.* (2015) genotyped 384 SNPs out of a total of 312,088 discoSNP++ predicted SNPs, of which 368 (95.8%) were accurately validated in the tick *Ixodes ricinus*.

**Table 4.** Single nucleotide polymorphism (SNP) and Indels in the *T. ilisha* genome.

SNPs / indels	Type	Number	%
Total SNPs	-	792939	100
Transitions	A>G	256209	32.31
	C>T	254042	32.04
Transversions	A>C	75912	9.57
	A>T	78469	9.90
	C>G	55810	7.04
	G>T	72497	9.14
Transition : Transversion	1.8 : 1		
Number of Indels	155574		

## Conclusions

We report here the first *de novo* genome of *T. ilisha* from the Bay of Bengal. The assembled genome can be used as a reference for genetic studies of *T. ilisha* and related species. The SNPs generated could provide a valuable resource for resolving stock disputes and phylogenetic or adaptation investigation of the Clupeidae family.

## Data Availability

### Underlying data

Tenualosa ilisha collected from Bay of Bengal, Accession number SAMN07556897: https://www.ncbi.nlm.nih.gov/biosample/?term=SAMN07556897

Tenualosa ilisha whole genome sequencing and assembly, Accession number SRP116260: https://trace.ncbi.nlm.nih.gov/Traces/sra/?study=SRP116260

Tenualosa ilisha whole genome shotgun sequencing project, Accession number SCED00000000: https://www.ncbi.nlm.nih.gov/nuccore/SCED00000000

Zenodo: Amino acid sequences of the proteins predicted from the whole genome of hilsa shad (Tenualosa ilisha) of the Bay of Bengal. https://doi.org/10.5281/ZENODO.2539223 ( Mollah *et al.,* 2019a)

Zenodo: Single Nucleotide Polymorphisms (SNPs) identified from the whole genome sequences of hilsa shad (Tenualosa ilisha) of the Bay of Bengal. https://doi.org/10.5281/ZENODO.2538155 ( Mollah *et al.,* 2019b)

Data are available under the terms of the Creative Commons Attribution 4.0 International license (CC-BY 4.0).
