# Supplementary material for: First draft genome assembly and identification of SNPs from hilsa shad ( Tenualosa ilisha) of the Bay of Bengal
Source: F1000Res. 2019 Mar 22;8:320. [Version 1] doi: 10.12688/f1000research.18325.1 (PMC6774053; doi:10.12688/f1000research.18325.1)
Supplement: Peer review history

# Peer review: Reviewer response for version 1

**DOI:** 10.5256/f1000research.20044.r53826

**Published:** 2019-10-01

**Authors:** Shotaro Hirase

**Affiliations:** 1 Fisheries Laboratory, Graduate School of Agricultural and Life Sciences, University of Tokyo, Hamamatsu, Japan

Authors constructed a draft genome assembly by whole genome sequencing and identified SNPs from *Tenualosa ilisha* of the Bay of Bengal. This paper is well written, and that the analysis methods are generally appropriate. I have several suggestions to improve this manuscript as follows:

1. Authors described that “We captured ten *T. ilisha *specimens from the seashore of the Bay of Bengal” in the Method section. But it is confusing because one individual was used finally in this study. Please revise this point.
2. Authors said that three different ecological types of hilsa shad have been recognized in Bangladesh. What kind of ecotype is the individual used for genome assembly expected to have? This information may be important for future studies focusing genomic bases of the ecotype divergence.
3. Authors described that “These results suggest higher completeness of the *T. ilisha *genome assembly of the Bay of Bengal.” It is a difficult problem whether it is higher completeness or not, but it seems that there are too many missing genes to say higher completeness.
4. SNP typing was performed with a reference-free method. However, I think that it would be better to detect SNP using the reference constructed newly in this study (mapping and SNP calling by samtools etc). The SNP information (e.g. vcf file) based on the constructed reference genome should be useful for readers.

I confirm that I have read this submission and believe that I have an appropriate level of expertise to confirm that it is of an acceptable scientific standard, however I have significant reservations, as outlined above.

# Peer review: Reviewer response for version 1

**DOI:** 10.5256/f1000research.20044.r47105

**Published:** 2019-05-21

**Authors:** Yuzine B. Esa

**Affiliations:** 1 Department Of Aquaculture, Faculty of Agriculture, University Putra Malaysia (UPM), Serdang, Malaysia

Overall, the manuscript was well written and contains all relevant information in accordance with its scope.

 Probably, it would be better if Table 3 is being presented in a figure form (e.g. Venn diagram), showing overlapping regions of genes between species.

 A linkage map (e.g using zebrafish as reference genome) and gene synteny analyses would be another interesting aspect that can be included.

 It would be more interesting if the genome of male and female of this species can be compared and analysed, since the fish is known as protandrous hermaphrodite.

I confirm that I have read this submission and believe that I have an appropriate level of expertise to confirm that it is of an acceptable scientific standard, however I have significant reservations, as outlined above.

## References

[1] : The draft genome of the grass carp (Ctenopharyngodon idellus) provides insights into its evolution and vegetarian adaptation. *Nat Genet*.2015;47(6) : 10.1038/ng.3280 625-31 10.1038/ng.3280 25938946

# Peer review: Reviewer response for version 1

**DOI:** 10.5256/f1000research.20044.r47164

**Published:** 2019-04-29

**Authors:** Joel A. Malek

**Affiliations:** 1 Genomics Core, Department of Genetic Medicine, Genomics Laboratory, Weill Cornell Medicine–Qatar, Doha, Qatar

The authors provide a new reference genome for the hilsa shad fish of significant commercial importance in the Bay of Bengal. The methods they use are standard and the resulting completeness analysis by BUSCO showed approximately 78% of core genes present. While it would be ideal to have added libraries for scaffolding the assembly (reads from 5-20kb inserts, or 10X Genomics reads, or Pacific Bioscience), this initial assembly will certainly allow some general comparisons and the building of genomic resources to better study the fish.

I confirm that I have read this submission and believe that I have an appropriate level of expertise to confirm that it is of an acceptable scientific standard.
